# Trust-building in the Pharmacist-patient Relationship: A Qualitative Study

**DOI:** 10.22037/ijpr.2020.114113.14675

**Published:** 2021

**Authors:** Rasool Esmalipour, Pooneh Salary, Amirahmad Shojaei

**Affiliations:** *Medical Ethics and History of Medicine Research Center, Tehran University of Medical Sciences, Tehran, Iran.*

**Keywords:** Trust-building, Pharmacist, Pharmacy, Pharmacy technician, Pharmacy’s management

## Abstract

A key element in therapeutic communication is trust and it needs to be created and maintained between health care providers and recipients reciprocally. This study aimed to identify the factors that can enhance and improve trust between pharmacists and patients. This study was a qualitative study consisting of an in-depth semi-structured interview followed by a focus group discussion. In the first phase of the study, a semi-structured open-ended interview was conducted with patients, pharmacists, and pharmacy technicians. The interview phase was followed by transcribing verbatim and content analysis and a focus group discussion. Finally, 49 items of trust-building factors between the patient and the pharmacists were obtained. A questionnaire was designed and distributed among 80 people for transparency and relevance, similar to the participants. The necessary corrections and changes were made in the items after collecting the answers. The study achieved two main themes; external and internal trust-building factors. Internal factors include the category of the factors related to human resources and managerial factors. Finally, 49 trust-building factors were developed. Internal factors are those factors in which the pharmacist, the pharmacy technician, and the pharmacy’s management system play a key role in building trust between pharmacists and patients.

## Introduction

Among health professionals, it is known that pharmacists have a lot of relationships and interactions with patients. In recent years, community pharmacies have emerged as a major group of health care providers (1). Pharmacists are considered as the first point of contact in primary health care (2). The role of community pharmacists in blood pressure control, which the Ministry of Health and Medical Education of Iran implemented in 2019, is an example of the community pharmacy being in a unique position. 

Although cyberspace and the internet provide a lot of information to the public, many people still refer to and trust pharmacists more than ever. Because they are the most accessible, reliable, and frequently visited providers of health care (2). The pharmacy profession like other health professions has undergone considerable changes due to scientific and technological advances and the provision of pharmaceutical care services by pharmacists has been considered as their professional responsibility that necessitates communication with patients. Accordingly, pharmacists must improve their ability to communicate professionally and effectively with patients to provide pharmaceutical care. Studies show that the relationship between pharmacist and patient is of the utmost importance, as it was observed by Salari *et al. * In this study performed for curriculum development, the relationship between pharmacist and patient obtained the highest score concerning its importance (3). 

One of the most important factors in establishing a good relationship between pharmacists and patients is trust, enabling an effective and useful relationship. Trust has a great impact on the effectiveness of treatment and satisfaction of patients which may influence the patient’s health status through continued care, adherence to the treatment process, and compliance (4). If we fully understand the meaning of trust, we may hope for its external realization and promotion. Trust achieves confidence, where a person can rely on another person’s genuine and moral competence, which in turn leads to an intimate and long-lasting relationship.

The role of physicians and pharmacists in the formation, strengthening, and preservation of trust and satisfaction in patients is very important (5), and it will bring in medical health care effectiveness (6). Therefore, establishing a trustful relationship between the pharmacist and patient will provide the patient with a sense of empowerment (1). Patients’ trust directly and positively affects the patients’ commitment to treatment (7). 

Unfortunately, in the past decades, the increased cost of medical services on the one hand and the behavior of some health professionals on the other, much damage has diminished the mutual trust between health care providers and patients (8). As stated in a strategic document by the country’s medical ethics board, a reduction in people’s trust in the medical community is a major threat (9). 

When the possibility of a loss of trust arises, the trust-building skills of the professionals are essential. When patients trust to health professionals diminishes, the number of claims due to medical errors rises. Therefore, designing and developing the strategies and tactics for trust-building and increasing patient satisfaction and loyalty to their health care providers, including pharmacists, seem necessary. For this purpose, the current study aims at finding factors to build trust and promote it in pharmacists-patients relationships.

## Experimental

*Methods*

This study is a qualitative, experimental study consisting of in-depth semi-structured interviews followed by content analysis with a conventional approach and focus group discussion performed in 2019 and 2020. Since the goal of the study was to gather various opinions from both inside and outside the pharmacies and the pharmaceutical system, we enrolled the pharmacists, pharmacy technicians, and patients who had been referred to the pharmacies. The interviewees were chosen by purposive sampling method recruiting the participants who were accessible. 

The interview guide was designed using ten open-ended questions for brainstorming on the topic and encouraging the participants to express their views within the framework of questions. The main issues which were considered in the design of the questions were quality and financial transparency of pharmaceutical services provided in pharmacies, the quality of pharmacy consultations, the impact of pharmaceutical rules and regulations and how they are performed, the appearance and dress code of pharmacy technicians, communication skills and the interaction between pharmacists and pharmacy technicians with patients, and the general atmosphere and lay-out of the pharmacy. 

The research team approved the interview guide after reviews and corrections. The interview guide was reviewed by a pharmacist, a pharmacy technician, and a patient for the interview guide to be valid. 

Each interview was appointed in advance. The interviews were recorded on two mobile phones and notes were written at the same time. The average interview duration was 40 min. The interviews were transcribed verbatim. The interviews continued until data saturation was reached and no new information was being generated. Accordingly, information saturation was obtained after 15 interviews, but 3 more interviews were conducted to ensure saturation. The total number of participants in the study was 18, and informed consent was obtained at the beginning of the interviews verbally. They were assured that any personal information would not be revealed to anyone except the research team, and the research team would adhere to confidentiality principles.

The transcriptions were checked in detail, word by word, and a number of them were returned to interviewees to be re-checked for more clarifications. The data analysis was performed with a general inductive approach. Data were encrypted, and the categories and subcategories were determined; similar codes and items were merged. After the final modifications and the achievement of trust-building factors in pharmacy, a focus group consisting of two medical ethics specialists, and a clinical pharmacist was formed, and themes, categories, subcategories, and codes were discussed to validate further and confirm the findings. Eventually, the number of items reached 50 which were used for questionnaire development. The questionnaire was distributed among 80 people, including pharmacists, pharmacy technicians, and patients as face-to-face or via email or WhatsApp media to assess the necessity, relevance, and transparency of the trust-building factors. Sixty participants filled the questionnaire completely (response rate = 75%). The validity of the questionnaire was evaluated by calculating the content validity ratio (*CVR*) and content validity index (*CVI*) to assess the necessity, relevance, and transparency of the items for trust-building. At this stage, no item was removed due to their scores and importance. Only two items were similar and merged, and some items required transparency, correction, and editing under the supervision of the research team. Finally, 49 items were generated.

## Results

Overall, 18 people were interviewed by semi-structured interviews in 2019. The demographic data of all interviewees were presented in Table 1. 

**Figure 1:**
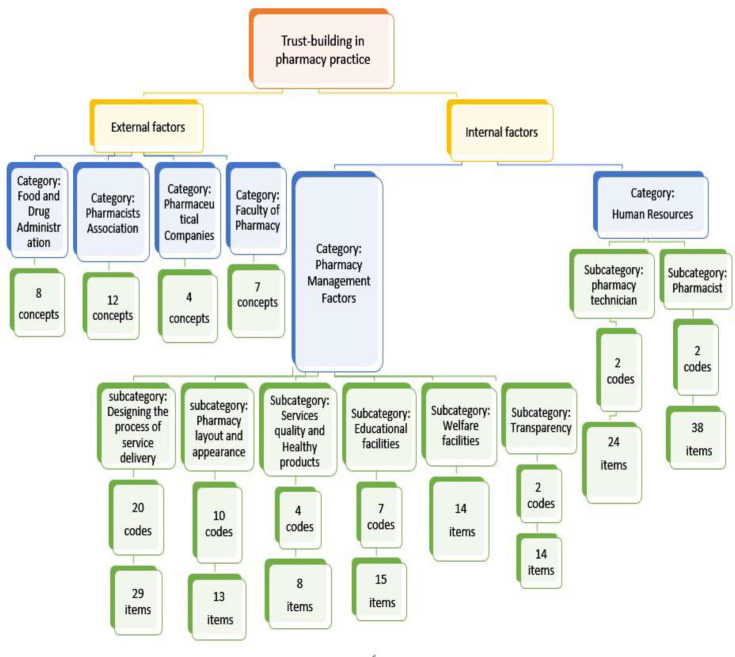
The concept map of the themes, Categories, Subcategories, and Codes

All categories and subcategories, and codes were presented in a concept map in Figure 1.

Generally, two themes called internal factors and external factors of trust-building in the pharmacy were attained.  

*External factors*

External factors consist of four categories related to the several elements outside of the pharmacy including the faculties of pharmacy, pharmacists’ associations, pharmaceutical companies, and the Food and Drug Administration. Some interviewees mentioned external factors of trust-building that had practical implications, but they are not modifiable by pharmacists and pharmacy technicians and cannot act upon. External factors that were highly emphasized were the role of faculties of pharmacy and pharmacists associations. In this regard, some of the participants stated:

*“... In my opinion, trust-building in the pharmacy should start from the basics, that is, from the faculties of pharmacy. Unfortunately, in the faculties of pharmacy, the pharmacy students do not learn how to communicate with patients acceptably, and solely scientific issues are taught. The students are never told that the main goal of healthcare is the patients’ health and wellbeing, and when the pharmacist graduates, his goal is to open a pharmacy and earn as much money as possible”*. (Pharmacist number 4)

*“.... Trust is generally a big, broad word, but I think two things can build trust. When a pharmacist association is talked about, at the first level, the independence of that association and at the second level the transparency are the things that can build trust. So the pharmacists’ associations play a key role here”. *(Patient number 5)

The external factors on building trust are not modifiable by the pharmacists. So, the external factors were left out to be investigated in other research in detail. 

*Internal factors*

The theme of internal factors consists of two categories: factors related to human resources and pharmacy managerial factors. 

The category of human resources is composed of two subcategories of pharmacist and pharmacy technician. The category of managerial factors includes subcategories of transparency in costs, welfare facilities, educational facilities, quality of services and health products, pharmacy layout and appearance and the process of providing pharmaceutical services. 

All interviewees mostly referred to the factors that can be implemented in the pharmacy environment by the pharmacist, pharmacy technicians, and in the management of the pharmacy to enhance trust-building. The interviewees emphasized communication skills as one of the most important factors of trust-building. They considered learning communication skills as necessary for all pharmacists; the interviewees indicated that pharmacists should establish their relationship with patients through all the senses by being a good listener, making good eye contact and body language. They recommended training communication skills at the beginning of pharmacy education. The participants stated: 

*“... One of the tasks of pharmacists is to listen to patients, which unfortunately is not taught in Iran. When the patient is not listened to as they should be, the patient/pharmacist trust is destroyed, and patients assume that the pharmacist just wants to make money and the patient is of no importance to them”. *(Pharmacist number 4)

*“... The pharmacist plays an important role in creating or not building trust by answering patients’ questions. For example, some pharmacists lack patience and respond to patients’ questions aggressively and out of impatience and anger which will, in turn, reduce a patient’s trust”. *(Patient number 1)

Some interviewees were concerned about the factors related to human resources; they referred to pharmacists’ consultations and mentioned that patients are usually unaware of what medications can be taken together, and even that they should be taken with food or not. They may even experience side effects if it is not well explained to them. When medicine that should be taken with food is taken on an empty stomach, it can cause burning and gastrointestinal pain. Another example is that patients should be warned and reminded when certain side effects can arise when taken together with other medications. A patient mentioned: 

*“... If there is a drug interaction in the prescription, pharmacists should make it known and advise me (the patient) on how to take medicines together, perhaps at intervals, or recommend alternatives. For example, should I take medicine with food or before eating?*

*In general, I should be guided better”.* (Patient number 4)

The layout of the pharmacy and patients’ privacy in this environment were considered very important in terms of the participants’ views, and considering it facilitates trust-building. This factor is deemed as one of the managerial factors of trust-building. The interviewees referred to the patients’ privacy and their personal information such as their identity, illness, medications, and the doctor’s name should be considered as very important and confidential as patients may be concerned. Furthermore, the patients may have private questions and are embarrassed to ask them in front of others. Patients prefer to go to a peaceful and quiet pharmacy and are humanely offered pharmaceutical consultation and care. The participants indicated:  

*“... Creating a good environment for patients where counseling and guidance are provided in a calm atmosphere is important, and privacy should be ensured and maintained, but there should not be a glass shield between the patient and the pharmacist”.* (Patient number 7)

*«…* *Another way to build trust is to have a counseling room. When the patient has personal questions, the pharmacist can easily answer the patient* *›* *s questions in a private room. If it is impossible, a special location can be allocated perhaps separate from other departments of the pharmacy, for example, by a partition, so that patients with* *‘* *embarrassing* *’* *questions can ask their individual questions, although still in the vicinity of others, and the pharmacist can provide the necessary advice and guidance there* *”.  *(Pharmacist number 4)

One of the managerial factors that the interviewees emphasized to a large extent and considered as the cause of a lot of mistrust was the lack of transparency in costs. In the interviews, they stated that patients are not aware of the price of medications because of the untimely and frequent changes in the price. According to the interviewees, although the patient may not complain about the price at the pharmacy, moral tension is generated and experienced by him as to why the cost of the medicine has increased so much. This subsequently creates negativity and mistrust in between. The interviewees considered that the solution to this problem is to provide a computer receipt to the patient where the medicinal items and their insurance conditions are stated. The pharmacy should provide explanations if the patient has more questions. Many misunderstandings will be prevented in this way, and the patient will continue his treatment process due to his confidence in the pharmacy and pharmacist. In this regard, a patient stated: 

*“... And one of the items that is very effective in trust building is to allocate enough time for transparency and explain financial issues to everyone where medical costs are shown item by item and how much the medications are covered by insurance and how much they are not. Everything should be clear to facilitate trust”*. (Patient number 7)

In agreement with the patient, a pharmacist said: 

*“…Presenting certain regulations and explanations about prices has a great effect on trust-building; even if it affects one out of 100 patients, it is still valuable. If the pharmacist determines the cost of medications and indicates how much is covered by insurance and how much must be paid in full, and give a full explanation, in that case, the pharmacist will be considered a good and trustworthy person”. *(Pharmacist number 4)

The content validity ratio (*CVR*) and content validity index (*CVI*) of the generated questionnaire were calculated and presented in Table 2. After merging two similar items and some corrections for increasing transparency, 49 internal trust-building factors were generated and presented in table 3. 

## Discussion

In this study, in searching into the trust-building factors in pharmacy and the pharmacists-patients relationship, we have reached two main themes: external and internal factors. External factors are related to the outside of the pharmacy environment, administrative, related to the pharmacists’ associations, or the educational system. The external factors are not modifiable by pharmacists and need more investigation. For example, Faculties of pharmacy as a part of the educational system and an external factor play a fundamental role in empowering pharmacists to perform professional duties. Eukel *et al.* stated that pharmacy students must acquire not only technical and clinical knowledge but also develop professional communications and behaviors in pharmacy schools to successfully perform patient-centered care as a pharmacist (10). Okoro observed that the educational intervention offered to final year students at the faculty of pharmacy in Nigeria improved their views on attitudes and behaviors related to professionalism and ethics in the pharmacy (11). 

Internal factors are related to the inside of the pharmacy environment, and pharmacists can implement their capabilities and those of the pharmacy technicians and managerial system in a way that builds trust or enhances it. According to our findings, internal factors were developed in two categories, including the factors related to human resources and managerial factors. One of the most important internal factors is the pharmacist, who can play a significant role, and his behavior is of utmost importance to patients (12). 

Based on the current study results, the factors related to human resources include two subcategories of pharmacist and pharmacy technician. Under the pharmacist subcategory, several factors were determined to have a fundamental role in building patients’ trust. Some of these factors represent professionalism and its principles. The pharmacist should be professionally committed to six principles of professionalism, including altruism, accountability, excellence, honor and integrity, respect for others, and justice (13, 14). 

Johnson and Chauvin indicated the critical role of professional commitment in trust-building in pharmacy (15). The results of our study present the important dimensions of the pharmacist’s professional behaviors, including honesty, listening carefully to the patient, respectful behavior, empathy, and being updated in both scientific and professional knowledge and skills. 

In this study, most pharmacists were dissatisfied with the lack of skill-based education in faculties. They considered that their professional behaviors were based on their personal experiences, religious and cultural beliefs. This problem exists in our country’s faculties of pharmacy as well as other countries, such as Sweden (12), and the importance of teaching principles of professionalism to pharmacy students was indicated previously (3). 

There are some occasions that the pharmacists-patient relationship is not coordinated and stable. Each one decides for the quality of the relationship and the process carryed out (16). The sign of good and effective communication is the establishment of understanding, and understanding implies the existence of perception and trust between the health professional and the patient. Therefore, if pharmacists establishe an effective and good relationship with patients, respect them, listen carefully to them, and behave honestly, they will be trusted by the patients (17). In this type of relationship that builds trust, the patient is considered as a part of therapeutic decision-making [18]. 

Other measures that have been obtained in our study are providing clear, sufficient, appropriate, and accurate pharmaceutical care and information. The results of this study show that the pharmacist should be careful in providing pharmaceutical consultations to patients and be sure that the patient understands and receives the information; otherwise, not only, the patient will not get a cure but also, he will be at risk of side effects. Providing pharmaceutical care is one of the most important responsibilities of a pharmacist. Hepler and Strand suggested that the pharmacy profession adopted the patient-centered and result-centered approach as its operating philosophy. This expanded professional role, known as pharmaceutical care, has been defined as «the responsible presentation of medication therapy to achieve definitive results that improve a patient›s quality of life» (19). 

One of the most important tasks in pharmaceutical care is to provide patient consultation that was found as a trust-building factor by our participants. Our findings are in agreement with other studies which emphasized allocating enough time to the patient, providing understandable answers to the patient’s questions, listening to the patient, and providing sufficient information as the trust-building factors between health professionals and patients (20). The issue of failure of proper counseling seems to be common, not only in our country but in all countries. Alte *et al.* observed that most northeastern pharmacies in Germany did not achieve their professional task in patient counseling. There is a need to improve the quality of counseling and diagnose drug-drug interactions (21). 

The pharmacy technicians who experimentally learn skills at the pharmacy are one group of human resources involved in pharmacy practice who play an important role in trust-building in some situations; they may even play a more important role than pharmacists. The pharmacy technicians should be familiar with communication skills because, in some cases, they are in direct contact with the patients, and their guidance and behavior affect the patients.

The way by which the pharmaceutical services are managed is another factor of trust-building that we have reached; however, the pharmacists’ role in managing the pharmaceutical care services is not fully understood in Iran unlike some developed countries (22, 23). 

The results of the current study show that several other managerial factors can play a role in trust-building, including transparency in the calculation of pharmaceutical costs and sufficient explanation about them which all participants emphasized. Therefore, the pharmacy managerial system should take measures to prevent misunderstandings which reduces patients’ trust, including printing the receipts containing the price of all medications and the insurance share price, as well as giving a full verbal explanation. Such issues can face the pharmacist with the dilemma of whether to act in the interests of the patient or the pharmacy because they are related to financial and economic affairs. 

Gidman *et al.* indicated that the economic aspects of pharmacies increased patients’ concerns about their loyalty. In this study, participants were concerned about the motivation and goals of pharmacists due to the economic context of the pharmacy which creates conflicts of interests and apprehension (24). 

Confidentiality and keeping the patients secret was a trust-building factor related to both pharmacist and the pharmacy technician indicated in “The Code of Ethics for the National Pharmaceutical System of Iran”. This code consists of eight principles derived from ethical principles and principles of professionalism that define several duties of pharmacists and are consistent with trust-building factors in the present study (25). 

The agreement between the results of our study and the other surveys shows that, firstly, the current study is on the right path. Secondly, the implementation of these factors has been emphasized by pharmacists and verified by codes of conduct. Therefore, it can be concluded that this group of professionals needs to properly familiarize themselves with ethical codes and try to implement them.

Also, the findings of this research are relatively consistent with what is performing by the Ministry of Health of Iran to evaluate the performance of pharmacies by two questionnaires; the pharmacy evaluation form and pharmacy inspection form. 

Since this study has determined trust-building factors based on the opinions of health care providers in the pharmaceutical system (pharmacist and pharmacy technicians) as well as the recipients of pharmaceutical care services (patients), it is feasible to claim that considering the obtained factors can create and maintain trust in an acceptable level.  

**Table 1:** Demographic data of the interviewees

**Interviewees**	**number**	**Age (year) (mean)**	**Work experience (year) (mean)**
Pharmacist	8 (including 6 PharmDs and 2 PhD holders in pharmaceutics)	45.5	17.8
Pharmacy technician	2	47.5	19.5
Patient	8	46	-

**Table 2:** *CVR* and *CVI* of the items of questionnaire

**Items**	***CVR***	***CVI***	**Items**	***CVR***	***CVI***
1	0.866	0.98	26	0.466	0.9
2	0.866	0.93	27	0.533	0.966
3	0.8	0.98	28	0.633	0.933
4	-0.3	0.833	29	0.7	0.933
5	0.43	0.933	30	0.533	0.9
6	0.8	0.933	31	0.866	3
7	0.633	0.966	32	0.6	0.95
8	0.13	0.866	33	0.6	0.966
9	0.4	0.91	34	0.33	0.966
10	0.666	0.98	35	0.533	0.966
11	0.9	0.98	36	0.9	0.966
12	0.166	0.866	37	0	0.95
13	0.966	0.98	38	0.733	0.98
14	0.8	1	39	0.7	0.933
15	0.833	0.966	40	0.933	0.98
16	-0.04	0.866	41	0.533	0.9
17	0.7	0.966	42	-0.4	0.666
18	0.866	0.98	43	0.4	0.9
19	0.366	0.933	44	0.033	0.81
20	-0.2	0.85	45	0.166	0.833
21	0.66	0.866	46	0.333	0.91
22	0.666	0.933	47	0.6	0.95
23	0.766	0.966	48	0.13	0.88
24	-0.2	0.85	49	0.56	0.95
25	0.233	0.783	50	0.633	0.95

*CVR*: Content validity ratio; *CVI*: Content validity index.

**Table 3:** Final trust- building factors

**Factors related to the pharmacist**
1- Being honest with patients
2- Listening carefully and patiently to patients
3- Being respectful
4- Empathizing with patients
5. Being familiar with the basics of communication skills
6- Knowing the legal responsibilities and rules of his profession
7- Having a neat and tidy appearance (professional appearance)
8- Wearing a white coat with name tag and title
9- Allocating enough time for each patient
10- Providing the necessary care and medical information correctly, appropriately, sufficiently and transparently
11- Answering the patient's telephone questions correctly, appropriately, sufficiently and clearly
12-Not supplying counterfeit, illegal and unlicensed medications
13. For medications with close expiry date, provides necessary explanations and instructions
14- Having a continuous and active presence in the pharmacy
15. Not having a financial relationship with the patient when providing services and medical care and consulting
16- Trying to update and improve scientific and professional skills
17. Respecting patients confidentiality
18. Managing situations of conflict of interest in an ethical manner
19. Giving good explanations about medication brands and giving patients enough time to choose their preferred medication from a variety of brands.
20. Being familiar with the basics of the Patients’ Rights Charter and respecting them
**Factors related to the pharmacy technician**
21. Being honest with patients
22. Empathizing with patients
23. Having a neat and tidy appearance
24. Wearing a white coat with name tag and title
25. Being respectful
26. Guiding patients correctly, honestly, sufficiently, accurately and clearly
27. Knowing the legal responsibilities and rules
28. Not interfering with the medications prescribed by the doctor and referring to the pharmacist if necessary
29. Respecting patients confidentiality
30. Trying to improve technical skills
31. Being familiar with the basics of the Patients’ Rights Charter and respecting them.
32. Being familiar with the basics of communication skills
**Factors related to pharmacy management**
33. Transparency in calculating costs and providing written invoices to patients
34. Legislation and adherence to regulations
35. Allocating suitable physical space for counseling and privacy
36. Choosing experienced and capable staff
37. Creating a fair process in the distribution of pharmaceutical resources and services
38. Not supplying counterfeit and illegal medications without license
39. Not supplying medications with a defective appearance, such as a broken tablet inside the blister
40.Putting up posters sent by the Food and Drug Administration in the right place and time
41. Installing the Patients’ Rights Charter for the public
42.Installing signs to introduce different parts of the pharmacy
43. Providing appropriate conditions for providing necessary counseling to patients in a scientific and ethical manner
44. Equipping the pharmacy with up-to-date and modern software and patient's electronic files
45. Preparing a procedure for contacting a physician if the name of the medication and its instructions are illegible
46. Inform patients about waiting time
47. Preparation of drug delivery process by pharmacist
48. Preparing the right process for the drug dispensing
49. Separating financial and professional activities

## Limitations

This study employed a limited number of urban pharmacies and their patients and did not include rural pharmacies due to the considerable challenges and limitations. In other words, different issues may arise due to the cultures of different regions, and these different cultures have their specific characteristics and require specific measures for trust-building; as an example, there are varying cultural views about privacy and confidentiality. Or there are different expectations between the people of provincial villages and cities regarding welfare and financial facilities. The position of pharmacies and pharmacists can vary significantly between rural and urban people. This issue can even vary from city to city. 

Another limitation was the allocation of time for interviews, as patients were often in a hurry to leave the pharmacy. These interviews were performed hurriedly. Therefore, the study does not claim to have achieved and developed all the trust-building factors in the pharmacy. The present study is a short study, and further research needs to be conducted in different areas and cities. Under these circumstances, the importance of awareness of the professional and legal standards and their training to health professionals, especially pharmacists and pharmacy technicians, is perceived more fully.

## Conclusion

To build trust in the pharmacist-patients relationship, pharmacists and pharmacy technicians should consider and commit to the internal factors for trust-building. These factors origins back to ethical principles and the principles of professionalism. Every pharmacist and pharmacy technician should fully perceive the message of this study to act to their duties more efficiently and more professionally. Doing this will revolutionize the patients’ adherence to treatment and patients’ compliance and will upgrade the pharmacists’ professional role in the health system. 

Furthermore, more investigations into the external factors are needed to explore the solutions by which we can empower the educational system and to make the policies more compatible with ethical principles and to build the infrastructures more friendly with the major responsibility of the pharmacists, providing pharmaceutical care. 
